# A patient with four metachronous cancers and multiple adenomatous colon polyps harboring the American Founder Lynch syndrome mutation: a case report

**DOI:** 10.1186/1897-4287-9-S1-P8

**Published:** 2011-03-10

**Authors:** Lindsay Dohany, Whitney Ducaine, Dana Zakalik

**Affiliations:** 1 Cancer Genetics Program, William Beaumont Hospital, Royal Oak, Michigan, USA

## Background

Lynch syndrome (LS) is a genetic disorder that accounts for approximately 3% of all colorectal cancers (CRC) [1]. Clinical characteristics of LS include proximal CRC, multiple CRCs, occurrence at a young age, accelerated carcinogenesis, and an increase in risk of extracolonic cancers [1]. LS is an autosomal dominant disorder, caused by germline mutations in DNA mismatch repair (MMR) genes. Mutations in *MSH2* account for 1-2% of all CRCs and up to 20% of these are large germline deletions [2]. The *MSH2* deletion of exons 1-6 has been characterized as a North American Founder Mutation (AFM) [2,3].

## Case report

A 68-year-old Caucasian male presented to cancer genetics following a second primary diagnosis of infiltrating poorly differentiated adenocarcinoma of the colon. His history included; moderately differentiated invasive adenocarcinoma of the sigmoid colon at age 40; left ureteral carcinoma diagnosed at 54; and a bladder carcinoma diagnosed at age 59. Additionally, colonoscopy revealed multiple adenomatous polyps within a ten year period. Family history is significant for a son diagnosed with colon cancer at age 34, father with gastric cancer, two paternal aunts and paternal grandfather with colon cancer, and German ancestry. Peripheral blood was sent for analysis of the *MLH1* and *MSH2* genes [4]. Molecular analysis identified a deleterious mutation, del exons 1-6, in the *MSH2* gene. This results in the premature truncation of the MSH2 protein and confirms the diagnosis of LS.

## Conclusions

This case reveals a LS patient with a history of four metachronous cancers. Phenotypic variations exist amongst the different MMR genes causative for LS [5-7]. Individuals with *MSH2* mutations are at a higher risk of developing extracolonic cancers than individuals with *MLH1* mutations [5,6]. There is a 7-fold higher risk for urinary tract cancers in individuals with *MSH2* mutations and male carriers have up to a 28% lifetime risk of developing uroepithelial cancers [8]. It is controversial whether or not large deletions lead to a more severe phenotype with multiple cancers and earlier ages of onset [7,9]. The severity of clinical presentation may correlate more with which MMR gene is altered than the specific mutation type [9]. A better understanding of genotype-phenotype correlations may allow for a personalized surveillance plan in the future.
